# Metabolic and Vascular Imaging Biomarkers in Down Syndrome Provide Unique Insights Into Brain Aging and Alzheimer Disease Pathogenesis

**DOI:** 10.3389/fnagi.2018.00191

**Published:** 2018-06-21

**Authors:** Elizabeth Head, David K. Powell, Frederick A. Schmitt

**Affiliations:** ^1^Department of Pharmacology & Nutritional Sciences, Sanders Brown Center on Aging, University of Kentucky, Lexington, KY, United States; ^2^Magnetic Resonance Imaging and Spectroscopy Center, Sanders Brown Center on Aging, University of Kentucky, Lexington, KY, United States; ^3^Department of Neurology, Sanders Brown Center on Aging, University of Kentucky, Lexington, KY, United States

**Keywords:** dementia, FDG-PET, hypometabolism, hypermetabolism, myoinositol, MR spectroscopy, T2^*^, trisomy 21

## Abstract

People with Down syndrome (DS) are at high risk for developing Alzheimer disease (AD). Neuropathology consistent with AD is present by 40 years of age and dementia may develop up to a decade later. In this review, we describe metabolic and vascular neuroimaging studies in DS that suggest these functional changes are a key feature of aging, linked to cognitive decline and AD in this vulnerable cohort. FDG-PET imaging in DS suggests systematic reductions in glucose metabolism in posterior cingulate and parietotemporal cortex. Magentic resonance spectroscopy studies show consistent decreases in neuronal health and increased myoinositol, suggesting inflammation. There are few vascular imaging studies in DS suggesting a gap in our knowledge. Future studies would benefit from longitudinal measures and combining various imaging approaches to identify early signs of dementia in DS that may be amenable to intervention.

## Introduction

The life expectancy of people with Down syndrome (DS) continues to increase due to improved health care and management of co-occurring illnesses (Bittles and Glasson, 2004). Consequently there are more people with DS and the population has grown from 49,923 in 1950 to 206, 336 in 2010 (de Graaf et al., 2017). However, mortality rate is higher in people with DS in older ages relative to the general population (Ng et al., 2017) and further, some deaths such as those due to respiratory disorders and epilepsy may be amenable to medical intervention (Hosking et al., 2016). As with the general population, the risk of developing health-related problems increases as people with DS get older. In particular, people with DS are at a high risk of developing cognitive impairment and clinical dementia after the age of 50 years (Zigman et al., 1996; Sinai et al., 2018). Virtually all adults with DS develop the neuropathology for a brain-based Alzheimer disease (AD) diagnosis by the age of 40 years (reviewed in Head et al., 2016). This is thought to be due to the lifelong overexpression of the APP gene on chromosome 21 leading to early onset and rapid accumulation of beta-amyloid (Aβ) with age (Head et al., 2016). Thus, by studying individuals with DS across the lifespan it is possible to identify early biomarkers of AD pathogenesis that may not be feasible in the general population as the age of onset of AD varies tremendously (e.g., 50-over 100 years). As will be discussed later in this review, cerebrovascular pathology may help to accelerate AD in DS and be an important contributor to dementia. Interestingly, a subset of adults with DS never develops dementia even in the presence of this AD pathology (Franceschi et al., 1990; Schupf and Sergievsky, 2002; Head et al., 2012a,b).

Neuroimaging studies help us detect structural, connectivity, activity, neurochemical, and vascular/metabolic functional changes with age and with the development of AD. As the development of AD neuropathology in DS is strongly age-dependent, we can learn about early changes associated with the progression of AD through neuroimaging studies in DS (Neale et al., 2018). These studies not only help us understand aging and AD in people with DS but can translate to our understanding of AD in the general population. For example, Jack and Holtzman (2013) proposed a hypothetical series of biomarker events including neuroimaging outcomes that may occur prior to changes in cognition and be predictive of decline in the general population. For example, Jack and colleagues suggest that Aβ can be detected prior to brain structure changes, which in turn are detectable prior to mild cognitive impairment and dementia. A similar series of biomarker events can be hypothetically applied for people with DS but using age as opposed to the clinical disease stage as the time axis. Whereas in the general population, biomarker changes reflecting progressive AD are plotted as a function of the cognitive continiuum, in DS we can use age as a representative to AD pathogenesis. It is clear from this hypothetical model that neuroimaging can provide early markers of dementia-associated brain changes and the inclusion of vascular or metabolic imaging may play an important role by providing even earlier information regarding AD pathogenesis.

## Metabolic imaging biomarkers—FDG-PET

Metabolic imaging using positron emission tomography (PET) has consistently shown reductions in glucose utilization in vulnerable brain regions in sporadic AD (Silverman and Phelps, 2001). In particular, the precuneus, posterior cingulate cortex and posterior parietotemporal lobes may be the earliest site of reduced glucose metabolism (FDG-PET) prior to onset of symptoms (Minoshima et al., 1997). In AD, the extent of cerebral metabolic rate of glucose from FDG-PET studies (CMRglu) is correlated with the severity of dementia (Minoshima et al., 1997). As AD progresses, more brain regions show declines in CMRglu such as the frontal cortex. CMRglu from FDG-PET can also predict AD neuropathology with 84–93% sensitivity and 73% specificity (Silverman et al., 2001; Jagust et al., 2007).

There are relatively few FDG-PET studies in DS with the majority being acquired under resting conditions (Table 1 summarizes studies since 1983). However the results of these studies have been relatively consistent. First, younger individuals with DS (without dementia) show increased glucose metabolism (Schwartz et al., 1983; Cutler, 1986; Azari et al., 1994a; Haier et al., 2003, 2008; Lengyel et al., 2006; Matthews et al., 2016) relative to age matched controls in all but one study (Schapiro et al., 1992b). The regions that show hypermetabolism include the prefrontal cortex, sensorimotor cortex, thalamus, inferior temporal/entorhinal cortex. Interestingly, increased glucose metabolic rate is associated with decreased gray matter volume in the temporal cortex including the parahippocampus/hippocampus suggesting that hypermetabolism is a compensatory response (Haier et al., 2008). Indeed, autopsy studies of the same brain region in a case series of DS shows evidence of neuronal sprouting positive for tau phosphorylation suggesting a mechanistic basis for increased glucose metabolism in middle age (Head et al., 2003). Other molecular events may also underlie this phenomenon (reviewed in Head et al., 2007).

**Table 1:** Summary of FDG-PET studies in DS (since 1983).

**Authors**	**Date**	**Resting or active**	**Cohort**	**Clinical data**	**Outcome**	**References**
Schwartz et al.	1983	Resting	*n* = 4 young DS 19–27 years and *n* = 1 aged DS 51 years, *n* = 10 young controls 21–33 years and *n* = 8 aged controls 45–55 years	None	Increased glucose metabolic rate in multiple brain regions in young DS relative to age matched controls whereas the 51 year old showed reduced metabolic rate to young DS controls (but not to old controls).	Schwartz et al., 1983
Cutler	1986	Resting	*n* = 10 DS 19–23 years, *n* = 4 DS 41–64 year (2 were demented) and *n* = 14 young 20–35 year and 19 40–60 year	None	Young and old control groups not different. CMRglu higher by 20–30% in young DS compared to young controls, lower CMRglu in old DS compared to young DS but not different from controls.	Cutler, 1986
Schapiro et al.	1988	Resting	*n* = 1 DS 47 years with autopsy confirmed AD, *n* = 13 non-demented DS, 19–33 years)	General intelligence, visuospatial ability, language and memory	Glucose metabolism was 28% less in the demented DS case involving the parietal and temporal cortex.	Schapiro et al., 1988
Schapiro et al.	1990	Resting	*n* = 14 non-demented DS 25–38 years, *n* = 13 controls 22–38 years.	PPVT	No differences in glucose metabolism in non-demented adults with DS relative to controls.	Schapiro et al., 1990
Horwitz et al.	1990	Resting	*n* = 14 DS < 34 years vs. *n* = 24 controls	None	The correlation between regional rates of glucose metabolism in DS was large and negative for the inferior frontal gyrus including Broca's area whereas in controls this association was positive.	Horwitz et al., 1990
Schapiro et al.	1992	Resting	*n* = 20 DS 19–34 years and 9 older DS, 45–63 years. Controls were *n* = 13 22 = 39 years.	Stanford-Binet, PPVT, Digit Span, Block Tapping Span and Object Pointing Span, Memory for Object subtest from DSMSE and recognition memory for designs, grammatic closure and manual expression, Hiskey Nebraska Block Pattery Subtest, WISC-R Block design	No difference in glucose metabolism found between young DS and young controls. DS with dementia had hypometabolism in association cortices (parietal and lateral temporal) and primary neocortex (sensorimotor and occipital). Mosaic/translation person with DS (45 years) with mild dementia showed reduced temporal glucose metabolism.	Schapiro et al., 1992a
Azari et al.	1994	Resting	*n* = 14 DS 26–38 years vs. *n*-17 controls	Stanford-Binet, DSMSE	DS vs. CTL not different on whole brain CMRglu, DS<CTL in left paracentral, left inferior parietal, anterior cingulate, posterior cingulate, right cerebellum, left cerebellum, DS>CTL left prefrontal, right sensorimotor, right thalamus.	Azari et al., 1994b
Azari et al.	1994	Resting	*n* = 15 Young DS, *n* = 10 older DS without dementia, *n* = 4 DS with dementia and *n* = 15 young controls	Stanford-Binet, DSMSE	All DS with dementia and some of the DS without dementia scans appeared similar to that of AD patterns.	Azari et al., 1994a
Dani et al.	1996	Resting	*n* = 12 non-demented DS 31–59 years, longitudinal evaluation—*n* = 2 (49,50 years) became demented	DSMSE	People with DS who were non-demented did not show changes in glucose metabolism over 7 years. Two people developed dementia after 7 years and showed a rapid decline in glucose metabolism in parietal and temporal regions.	Dani et al., 1996
Pietrini et al.	1997	Resting and audiovisual stimulation	*n* = 8 DS 32–38 years and *n* = 8 DS 43–61 years	Stanford-Binet Intelligence Scale, DSMSE, PPVT	At rest, no differences in glucose metabolism were observed. During stimulation, older subjects with DS showed reduced glucose metabolic rates in parietal and temporal cortex.	Pietrini et al., 1997
Haier et al.	2003	Continuous performance task	*n* = 17 DS (non-demented 34–55 years), *n*- *n* = 10 moderate AD, *n* = 12 young controls, *n* = 12 old controls)	Wechsler Adult Intelligence Scale III, DSDS, DMR, MMSE (for AD)	DS and AD participants had lower glucose metabolic rates relative to each of their control groups in the posterior cingulate. DS had higher GMR in inferior temporal/entorhinal cortex where AD subjects had lower GMR. Non-demented people with DS showed hypermetabolism as a compensatory response.	Haier et al., 2003
Lengyel et al.	2006	Resting	*n* = 11 DS (5–24 years) and *n* = 9 control (4–24 years)	None	Glucose metabolic rate was higher in DS relative to controls in 6 regions: left medial temporal gyrus, left precentral and inferior frontal gyri, anterior cingulate gyri.	Lengyel et al., 2006
Haier et al.	2008	Continuous performance task	*n* = 19 non-demented DS 34–52 years	DMR	Higher glucose metabolic rate was associated with decreased gray matter volume in temporal cortex including the parahippocampus/hippocampus, in the thalamus, caudate, and frontal lobe. Results consistent with a compensatory response.	Haier et al., 2008
Rafii et al.	2015	Resting	*n* = 12 non-demented DS (30–60 years)	CANTAB, RBANS, VABS, Observer Memory Questionnaire-Parent Form, Anxiety Depression and Mood Scale, Cambridge Examination for Mental Disorders of Older People with Down's syndrome and other with Intellectual Disability, Dalton Dyspraxia scale, Goodenough-Harris Draw-A-Person Test	People with DS over 39 years age of showed consistent hypometabolism by clinical read. No correlations with cognition.	Rafii et al., 2015
Sabbagh et al.	2015	Resting	*n* = 5 DS with AD, 12 non-demented DS and 9 normal controls	DMR, MMSE, BPT, SIB and VABS	DS with AD showed hypometabolism in posterior cingulate, lateral parietal, and temporal and frontal regions. Non-demented and demented DS had lower glucose metabolic rates in additional frontal regions compared with controls.	Sabbagh et al., 2015
Matthews et al.	2016		*n* = 12 non-demented DS (32–61 years), *n* = 12 normal, *n* = 12 early MCI, *n* = 12 late MCI, *n* = 12 AD and *n* = 12 and controls from ADNI	VABS	Posterior cingulate cortex and hippocampus showed hypometabolism in DS relative to controls. Lower glucose metabolic rate was observed in regions showing volumetric losses (mid cingulate, anterior cingulate, paracentral lobule, and hippocampus). Hypermetabolism was observed with preserved volume in the prefrontal cortex but reduced volume in occipital cortex. Patterns of glucose metabolism and volume losses corresponded to amyloid burden (florbetapir) and cognition.	Matthews et al., 2016
Rafii et al.	2017	Resting	*n* = 12 non-demented DS 30–60 years, *n* = 12 normal older adults, *n* = 12 AD	CANTAB, RBANS, VABS, Observer Memory Questionnaire-Parent Form, Anxiety Depression and Mood Scale, Cambridge Examination for Mental Disorders of Older People with Down's syndrome and other with Intellectual Disability, Dalton Dyspraxia scale, Goodenough-Harris Draw-A-Person Test	Areas with lower glucose metabolic rates were associated with tau accumulation by PET (F-AV-1451).	Rafii et al., 2017
Lao et al.	2018	Resting	*n* = 16 non-demented DS (Avg age 35 years—PiB negative), *n* = 5 non-demented DS (Avg age 47 years—PiB positive), *n* = 3 demented DS (Avg age 49 years)	PPVT	Glucose metabolism was reduced in the parietal cortex with increased PiB (amyloid) binding. Glucose metabolism was negatively associated with age (frontal, parietal, and temporal cortex). No correlation between striatal binding for PiB (amyloid) and glucose metabolism.	Lao et al., 2018

*CMRglu, glucose cerebral metabolic rate; GMR, glucose metabolic rate; PPVT, Peabody Picture Vocabulary Test; DSMSE, Down syndrome mental state examination; WISC, Wechsler Intelligence Scale for Children; DSDS, Dementia scale for Down syndrome; DMR, dementia questionnaire for mentally retarded persons; RBANS, Repeatable Battery for the Assessment of Neuropsychological status; CANTAB, Cambridge Neuropsychological Test Automated Battery; VABS, Vineland Adaptive Behavior Scale; BPT, Brief Praxis Test; SIB, severe impairment battery*.

In contrast, older individuals with DS and particularly those with dementia show hypometabolism in multiple studies (Schwartz et al., 1983; Cutler, 1986; Schapiro et al., 1992a; Azari et al., 1994a,b; Rafii et al., 2015; Sabbagh et al., 2015; Matthews et al., 2016). Brain regions that appear to be systematically affected under either resting or active conditions include the posterior cingulate cortex, hippocampus, parietal and temporal cortex consistent with reports in sporadic AD (Minoshima et al., 1997; Pietrini et al., 1997; Silverman and Phelps, 2001). Further, in a 45 year old female with mosaic/translation DS with clinical signs of early dementia, a pattern of hypometabolism similar to that of sporadic AD was observed (Schapiro et al., 1992a).

Reduced glucose metabolism in older adults with DS and dementia is associated with decreased cortical volumes (Matthews et al., 2016), increased amyloid binding with florbetapir (Matthews et al., 2016) and increased tau binding using AV-1451 (Rafii et al., 2017). Some studies report associations between cognition and glucose metabolism (Haier et al., 2008; Sabbagh et al., 2015; Matthews et al., 2016) but not all (Rafii et al., 2015), with variable results likely due to smaller sample sizes. In one of the only longitudinal studies that was found, Dani and colleagues reported stable glucose metabolic rates over a 7 year period of time unless clinical dementia had evolved (occurred in one person with DS) leading to rapid glucose metabolic decline in parietal and temporal cortices (Dani et al., 1996).

Reductions in glucose metabolism may lead to or reflect neuronal loss, synapse loss, and/or mitochondrial dysfunction. Given that all these events are thought to occur with age and dementia in DS (Head et al., 2016), PET imaging can provide useful information with respect to brain function but there is a clear need for more longitudinal studies that includes measures of cognition. It is also notable that despite the posterior cingulate cortex being an early site of glucose metabolic losses, there are few studies of AD neuropathology in this region in DS. The use of FDG-PET to capture information about metabolism requires the use of intravenous injections of radioactive ligands. This procedure may be problematic for some participants, their families and particularly for those with dementia. However, as an outcome measure that may reflect a rapid response to treatment that is targeting metabolism, FDG-PET has utility. In future, similar outcomes reflecting metabolic changes such as blood flow, may be obtainable using relatively short MR protocols such as arterial spin labeling (7 min). Further, as will be discussed next, magnetic resonance spectroscopy, which is also a relatively short protocol (5 min) that may be useful for a broader spectrum of participants can provide specific metabolic markers that could help dissect the different neuronal/glial pathways that signal onset of dementia.

## Metabolic imaging biomarkers—MRS

Proton magnetic resonance spectroscopy (^1^H-MRS) has been widely used to characterize the neurochemistry of brain health and disease. In particular, the neuronal markers of N-acetylaspartate (NAA) and glutamate-glutamine complex (Glx) decrease, and the glial marker of myo-inositol (MI) increases, both correlate with clinical variables in aging and AD (Parnetti et al., 1997; Lin and Rothman, 2014). It is thought that lower levels of NAA or Glx reflects neuronal loss or injury; neuroinflammation is associated with activated astrocytes and microglial cells leading to increased MI (Chang et al., 2013). The ratio of NAA to MI can also be used to distinguish non-demented from demented people (cf. Lin et al., 2005).

In DS, there have been several studies using MRS with assessments done for posterior cingulate cortex, hippocampus, frontal cortex, occipital cortex, and parietal cortex with comparisons to age matched controls (Table 2). Decreased NAA and increased MI is observed relatively consistently across studies in non-demented adults with DS compared with age matched non-DS controls (Shonk and Ross, 1995; Berry et al., 1999; Huang et al., 1999; Beacher et al., 2005; Lamar et al., 2011; Lin et al., 2016) with a few exceptions (Murata et al., 1993; Smigielska-Kuzia et al., 2010). Hippocampal Glx was not different in people with DS from controls (Tan et al., 2014). It may not be surprising that MI levels are generally higher in people with DS as the MI cotransporter (SLC5A3) gene is on chromosome 21 and is overexpressed in DS (Berry et al., 1995). Further, synaptojanin 1 (gene also on chromosome 21) can lead to increased gliosis (Herrera et al., 2009) and thus possibly, higher MI levels.

**Table 2:** Summary of MRS studies in DS (since 1993).

**Authors**	**Date**	**Brain Region**	**Cohort**	**Clinical data**	**Outcome**	**References**
Murata et al.	1993	white matter around the anterior horn of the lateral ventricle of frontal cortex	*n* = 18 DS 20–40 years, *n* = 15 controls (avg age 35.8 years)	Koh's Block-Design Test, Goodenough's Draw a Man Test	No differences in NAA noted across the groups nor as a function of age. MI was not analyzed. Note, many of these DS participants were institutionalized and controls were physicians and staff members. Two people with DS were mosaic.	Murata et al., 1993
Shonk & Ross	1995	N/A	*n* = 23 non-demented DS (age unknown), *n* = 1 demented DS (age unknown)	Unknown	MI was elevated in non-demented people with DS relative to controls and further elevated in DS with dementia. NAA was decreased in the DS with dementia participant.	Shonk and Ross, 1995
Huang et al.	1999	parietal and occipital cortex	*n* = 8 young DS 28–39 years, n-11 non-demented older DS 42–62 years, *n* = 8 young controls 22–39 years and *n* = 9 old controls 40–64 years	None	MI was 50% higher in occipital and parietal cortex of adults with DS relative to controls. Older DS subjects had higher MI than younger DS subjects. NAA was lower in older DS and controls relative to younger controls but not exacerbated in DS.	Huang et al., 1999
Berry et al.	1999	corpus striatum	*n* = 14, 1.2–13.6 years DS and *n*-13 1.3–11.7 year old controls	None	MI was significantly higher (28%) in basal ganglia of DS relative to controls. Choline containing compounds were significantly lower in DS relative to controls.	Berry et al., 1999
Beacher et al.	2005	Hippocampus	*n* = 38, 18–66 years non-demented DS and *n* = 42, 19–66 controls	CAMCOG	Hippocampal MI was higher in DS than controls. In DS, hippocampal MI correlated with cognition.	Beacher et al., 2005
Smigielska-Kuzia et al.	2007	Frontal cortex	*n* = 14 non-demented DS 7–17 years, *n* = 20 controls	None	Signficantly decreased Glx and NAA observed in DS relative to controls.	Smigielska-Kuzia and Sobaniec, 2007
Smigielska-Kuzia et al.	2010	Temporal lobes	*n* = 20 DS 3–15 years and *n* = 20 controls 6–15 years	None	Glx was not different between the DS and control groups. NAA and MI was significantly lower in DS relative to controls. GABA was also lower in DS.	Smigielska-Kuzia et al., 2010
Lamar et al.	2011	Hippocampus	*n* = 35 demented people with DS (Avg age = 52.8 years), *n* = 18 non-demented DS (Avg age 47.2 years) and *n* = 13 controls (Avg age 50.6 years), *n* = 39 sporadic AD (Avg age 76.8 years)	CAMDEX	MI was significantly higher in DS with dementia relative to non-demented DS (13% higher) and controls (19% higher). NAA was significantly reduced in DS with dementia relative to DS without dementia (11%) but was similar to controls. DS with dementia MI was significannlty higher relative to sporadic AD.	Lamar et al., 2011
Tan et al.	2014	Hippocampus	*n* = 35 non-demented DS (Avg age 35 years), *n* = 11 demented DS (Avg age 52 years) and *n* = 39 controls (Avg age 35 years)	CAMCOG	No differences in Glx across the 3 groups nor correlation with cognition.	Tan et al., 2014
Lin et al.	2016	Posterior Cingulate Cortex	*n* = 22 non-demented DS (Avg age 46.7 years), *n* = 5 demented DS (Avg age 53.7 years), *n* = 15 control (Avg age 47.8 years)	SIB, BPT, DMR	NAA reduced in DS with dementia relative to controls and non-demented DS groups. MI increased in DS relative to controls but not further increased with DS and dementia. Higher NAA levels associated with cognition (i.e. better performance associated with more NAA).	Lin et al., 2016

*DMR, dementia questionnaire for mentally retarded persons; BPT, Brief Praxis Test; SIB, severe impairment battery; CAMDEX, Cambridge Mental Disorders of the Elderly Examination; CAMCOG, Cambridge Cognitive Examination*.

With age, older people with DS show higher MI and lower NAA than younger people with DS. MI was higher in the occipital and parietal cortex of older DS subjects relative to younger people with DS (Huang et al., 1999). In the hippocampus of older adults with dementia with DS, MI is also higher and NAA lower when compared to non-demented people with DS (Lamar et al., 2011) but Glx is unchanged (Tan et al., 2014). In the posterior cingulate cortex, MI was not significantly different in people with DS who were demented compared with those who were not demented, but NAA was significantly decreased (Lin et al., 2016). However, there is a case report of an individual with DS who was demented showing higher MI and lower NAA relative to non-demented DS individuals (Shonk and Ross, 1995). Further increases in MI reported in some studies with aging and dementia may reflect increased neuroinflammation that has been observed with aging in DS (Wilcock, 2012).

Thus, MRS provides novel information and unique signatures for DS (e.g., higher MI) but also may be amenable to future treatment studies as metabolic outcomes measured by MRS may be rapidly modifiable as opposed to outcomes reflecting brain structure. Comparing MRS outcomes in different affected brain regions in people with DS (e.g., comparing hippocampus, frontal cortex, cingulate cortex) may provide novel links between the presence of *in vivo* amyloid by PET imaging and glial/neuronal consequences. For example, as amyloid PiB binding increases with age, how does NAA or MI decrease or increase correspondingly? These studies may lead us to novel interventions in future for DS with outcome measures and a further examination of the link between MRS outcomes, brain region, and cognition will be useful in future.

## Vascular imaging biomarkers

Cerebrovascular pathology occurs in over 85% of autopsy cases presenting with AD neuropathology and is associated with impaired cognition (Arvanitakis et al., 2016). One form of this pathology, cerebral amyloid angiopathy (CAA) is present in near all brains of people with AD (Viswanathan and Greenberg, 2011). Thus, there is an increasing recognition that along with the development of Aβ plaques and neurofibrillary tangles, vascular neuropathology may also affect cognition and the progression of dementia (White et al., 2002). Interestingly, in DS, there is significant cerebrovascular neuropathology in the form of CAA, primarily due to the overexpression of APP and Aβ (Ikeda et al., 1994; Iwatsubo et al., 1995; Mendel et al., 2010; Head et al., 2017; Zis and Strydom, 2018).

Extensive CAA is associated with microhemorrhages and strokes in general (Arvanitakis et al., 2017; Banerjee et al., 2017) although in DS, stroke is relatively rare (Buss et al., 2016). Nonetheless, CAA may have a significant impact on blood vessel function. CAA can lead to deficits in cerebrovascular regulation (Grinberg et al., 2012) and reduced blood flow may lead to impaired perivascular clearance of Aβ. Impaired clearance will in turn lead to additional accumulation of Aβ (Banerjee et al., 2017).

Neuroimaging of CAA is typically by GRE or T2^*^-weighted MRI (Fazekas et al., 1999). There is only one neuroimaging study using T2^*^ to observe the extent of CAA *in vivo* in older adults with DS (Carmona-Iragui et al., 2017). CAA was observed in 31% of cognitively impaired people with DS, which is similar to early onset AD (38%) and higher than sporadic AD (12%). In addition, 15.4% of people with DS had evidence of intracerebral hemorrhages. Thus, CAA is a consistent feature of aging and dementia in DS and may serve as a future target for clinical trials.

While PET-based studies in DS show metabolic differences that mirror AD in the general population, changes in blood flow may also be seen in DS. For example, single photon emission computed tomography (SPECT) patterns in younger individuals with DS reveal perfusion changes in temporal, parietal, and occipital regions (Kao et al., 1993) that are also reminiscent of those seen in AD (DeKosky et al., 1990). However, these regional differences in perfusion may reflect the added impact of CAA-associated or other cerebrovascular mechanisms in DS.

**Figure 1:**
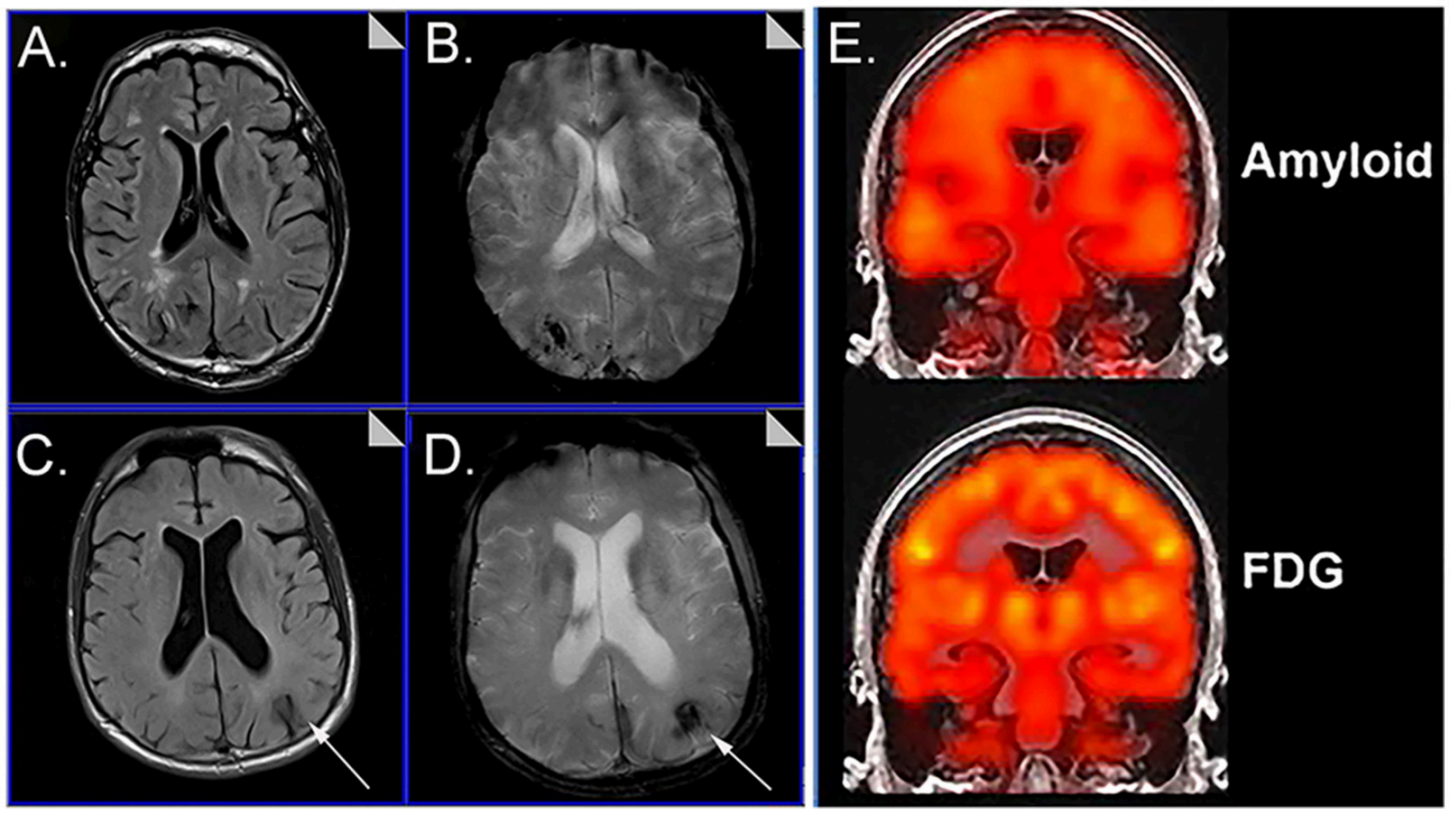
Representative examples of neuroimaging protocols acquired in Down syndrome. Panels **(A–D)** show MR imaging in a 57 year old male and 59 year old male imaged with T2^*^ **(A, C)** and FLAIR **(B, D)** showing the presence of microbleeds. Arrows distinguish edema and hemosiderin positive microbleeds. Panel **(E)** shows examples of amyloid PiB-PET imaging (top) and FDG-PET imaging (bottom). Images from **(A–D)** were modified from Figure 5 with permission of Elsevier Press (Head et al., 2018). Images from **(E)** were modified from Figure 1 reproduced with permission from Dr. M. Rafii and under the Creative Commons Attribution License (CC BY) (Rafii et al., 2015).

Cerebrovascular dysfunction measured *in vivo* may be critical for understanding not only the aging process and progression to AD in DS but treatment that rely on and are also relatively short MR protocols (T2^*^~7 min, FLAIR~4.5 min) (Figure 1). Immunotherapy trials in patients with AD suggest that cerebrovascular adverse effects can occur and are visualized with FLAIR (Sperling et al., 2012). The possibility of a similar outcome in DS is as yet unknown. Intervention studies that target Aβ or other pathways may be less effective in people with DS with significant cerebrovascular pathology and can confound the opportunity to observe benefits in clinical trials. Characterizing the extent of cerebrovascular pathology may serve as an exclusion/inclusion criteria or included as a covariate so as not to obscure positive clinical outcomes.

## Developmental differences and caveats with neuroimaging in DS

Structural differences in childhood and early adulthood suggest that some brain regions (e.g.,) are smaller in people with DS whereas others (parahippocampal gyrus) may be larger (Kesslak et al., 1994; Teipel and Hampel, 2006). It will be important to consider additional volumetric tissue losses using structural MRI when interpreting reductions in vascular flow or metabolic outcomes. Additional atrophy occurs with aging in DS and with the development of dementia with posterior cingulate, parietal, temporal, and frontal regions being affected (summarized nicely in Neale et al., 2018).

In studies of people with DS, sample sizes are typically smaller. This is due to challenges with recruitment, the ability of people to be scanned (e.g., fear) or to stay motionless (movement artifacts, people with dementia; Neale et al., 2018). Obesity or being overweight can lead to discomfort in the scanner and in some cases, may preclude a person from participating. The neck and facial structure of people with DS also can lead to discomfort in the prone position. For these situations, there are methods to provide additional padding and support along with frequent pauses in the procedures. Unfortunately, in many studies this leads to small sample sizes of demented individuals with DS, which may skew our results. Anxiolytics can be helpful when obtaining structural images but may interfere with functional measures. In our own experiences, we have found that repeated visits leads to greater successes with our volunteers participating in the scanning procedures and the option of anxiolytics has been helpful. Age of the participant also influences success with scanning. Estimates from our cohort suggest that a full set of images (MPRAGE, FLAIR, T2^*^, ASL, MRS, DTI ~50 min), 92% of people 25–37 years, 82% of 37–50 year olds and 40% of 50–65 year olds can be successfully scanned (unpublished observations from the University of Kentucky Down syndrome and Aging study). However, there are fewer sets of full imaging protocols we can acquire with increasing age as our participants may not be able to stay in the scanner as long as we require.

## Summary and future directions

Longitudinal studies in virtually all of the imaging parameters discussed here are critical. There are few longitudinal studies of metabolic and vascular neuroimaging changes with age in DS. In studies of structural imaging some show progressive atrophy (reviewed in Neale et al., 2018). Over a 3 year period of time, studies in non-demented adults with DS report an increasing number of individuals developing amyloid by PET (PiB binding) and those with existing amyloid binding showed an increasing number of brain regions affected along with increased accumulation within affected brain regions (Hartley et al., 2017; Lao et al., 2017; also reviewed in Neale et al., 2018) (Figure 1). It is interesting to note that PiB tends to bind more mature amyloid deposits (LeVine et al., 2017) *in vitro* consistent with the typical age of onset of PiB binding in DS being after 40 years of age. In summary, neuroimaging is a powerful tool to detect structural, metabolic and vascular changes with age and dementia in DS but there are still important gaps in our knowledge remaining. Feasibility concerns may be overcome with the use of mock scanners, increasing sample sizes (based upon estimates of scan success as a function of age and dementia) and reducing scan times. Given that neuroimaging outcomes could be critically important in future clinical trials, it will be important to encourage further studies for people with DS.

## Author contributions

All authors listed have made a substantial, direct, and intellectual contribution to the work, and approved it for publication.

### Conflict of interest statement

The authors declare that the research was conducted in the absence of any commercial or financial relationships that could be construed as a potential conflict of interest.
